# Influence of stem undersizing during direct anterior approach for total hip arthroplasty on periprosthetic bone biomechanics: a finite element analysis

**DOI:** 10.1302/2046-3758.1511.BJR-2026-0175.R1

**Published:** 2026-11-12

**Authors:** Jakub Liskay, Petr Marcián, Vasileios Apostolopoulos, Luboš Nachtnebl, Tomáš Tomáš

**Affiliations:** 1 First Department of Orthopaedic Surgery, St. Anne’s University Hospital and Faculty of Medicine, Masaryk University, Brno, Czechia; 2 Institute of Solid Mechanics, Mechatronics and Biomechanics, Faculty of Mechanical Engineering, Brno University of Technology, Brno, Czechia

**Keywords:** Direct anterior approach, Femoral stem undersizing, Finite element analysis, direct anterior approach, Total hip arthroplasty (THA), biomechanics, strains, cancellous bone, femoral stems, trabecular bone, cementless total hip arthroplasty, proximal femur, bone tissue

## Abstract

**Aims:**

Stem undersizing during cementless total hip arthroplasty (THA) may occur more frequently when the procedure is performed through the direct anterior approach (DAA) because of limited femoral exposure. Insufficient stem–bone contact may compromise primary stability and alter load transfer in the proximal femur. The aim was to investigate the biomechanical effects of femoral stem undersizing on periprosthetic bone using finite element analysis. A secondary objective was to evaluate whether the presence of a femoral stem collar modifies these biomechanical effects.

**Methods:**

Patient-specific finite element models of the full femur were generated from CT data. Eight finite element models were analyzed, combining four implantation configurations of the AMIStem-P designs (Adequate Fit, Adequate Fit–Collared, Undersized, and Undersized–Collared) with two cancellous bone density conditions, representing 100% and 90% cancellous bone density. A hip joint force representing the peak loading phase during normal walking/single-leg support was applied. Strain intensity in periprosthetic trabecular bone was assessed according to Frost’s mechanostat theory, and von Mises stress distribution in the femoral stem was evaluated.

**Results:**

Stem undersizing markedly increased strain intensity in the periprosthetic trabecular bone and enlarged regions exceeding the mechanostat overload threshold (> 3,000 µε). Adequate stem–bone fit with cortical support substantially reduced the volume of overloaded bone tissue. The presence of a collar reduced strain levels, particularly in undersized configurations, indicating improved proximal load transfer. Reduced cancellous bone density resulted in a moderate additional increase in strain. Simulated load reduction markedly decreased periprosthetic strain levels. Implant stresses remained well below material limits.

**Conclusion:**

Femoral stem undersizing may substantially increase strain within the periprosthetic trabecular bone and enlarge regions exposed to overload conditions. Achieving adequate stem–bone fit with cortical support was associated with a marked reduction in periprosthetic bone strain. The presence of a collar appeared to partially mitigate the adverse mechanical effects of undersizing by improving proximal load transfer.

Cite this article: *Bone Joint Res* 2026;15(11):1354–1363.

## Article focus

- To investigate the biomechanical effects of femoral stem undersizing on periprosthetic bone strain distribution in cementless total hip arthroplasty.
- To evaluate whether the presence of a femoral stem collar mitigates the adverse mechanical effects of undersizing.
- To assess the influence of reduced cancellous bone density on periprosthetic load transfer.

## Key messages

- Femoral stem undersizing substantially increased periprosthetic trabecular bone strain, particularly in regions exceeding the mechanostat overload threshold (> 3,000 µε).
- Adequate stem–bone fit with cortical support reduced excessive strain, while the presence of a collar partially mitigated the adverse biomechanical effects of undersizing.
- Simulated load reduction markedly decreased periprosthetic bone strain, suggesting a potential biomechanical benefit of protected postoperative weightbearing when primary fixation is compromised.

## Strengths and limitations

- Finite element analysis enabled a systematic comparison of eight model configurations, evaluating the effects of stem sizing, collar presence, and cancellous bone density on periprosthetic bone biomechanics.
- The study was limited to a single femoral geometry and one femoral stem design family, potentially restricting the generalizability of the findings.
- Simplified loading and implant–bone interface conditions did not account for dynamic activities or biological fixation, preventing direct prediction of clinical outcomes.

## Introduction

Total hip arthroplasty (THA) using the direct anterior approach (DAA) has gained popularity in recent years due to perceived advantages such as muscle-sparing dissection, reduced postoperative pain, and expedited functional recovery compared with traditional approaches.^1^ However, this minimally invasive technique is also associated with a unique set of challenges that stem from its limited surgical exposure and technical demands. The DAA’s constrained visual and instrumental access to the proximal femur can complicate femoral preparation, particularly in cementless implantations, and may predispose to femoral component undersizing, especially during a surgeon’s early experience with the approach.^2,3^ The clinical relevance of this issue is supported by registry data showing that the DAA had higher revision rates for loosening and early fracture compared with posterior and lateral approaches.^4^ In addition, a recent registry analysis of 48,567 DAA THAs reported a higher early revision risk with collarless cementless stems compared with collared stems, supporting the relevance of stem fixation and collar support in this setting.^5^ Stem undersizing should not be regarded as a problem unique to the DAA, because inadequate stem–bone contact, reduced metaphyseal or cortical engagement, early migration, and subsidence may occur in cementless THA performed through different surgical approaches.^6-9^ Therefore, the DAA in the present study should be interpreted as a clinically relevant scenario.

Undersizing of cementless femoral stems has been associated with several detrimental biomechanical and clinical consequences that may compromise implant success.^3^ In short-stem and conventional cementless THA, stems that do not achieve adequate contact with the lateral femoral cortex – often a hallmark of undersizing – demonstrate significantly greater axial migration compared with appropriately sized components, suggesting reduced primary stability, a key determinant of long-term fixation success.^10,11^ In one clinical series, stems that were two sizes smaller than planned exhibited increased migration at mid-term follow-up, reflecting insufficient mechanical engagement with the host bone.^11^ In controlled cadaveric experiments, undersized stems displayed substantially greater subsidence and lower resistance to extraction forces, underlining the risk that inadequate sizing impairs the mechanical environment necessary for stable bone–implant contact.^10^

Despite mounting evidence that stem undersizing can undermine primary stability and alter mechanical performance in cementless THA, there remains a notable gap in the literature with respect to how sizing discrepancies influence periprosthetic bone biomechanics, particularly when surgery is performed through the DAA. While finite element (FE) studies have examined factors such as interference fit and primary micromotion at the implant–bone interface, as well as the impact of design parameters on fracture load and stress distribution, few have directly addressed the specific effect of undersizing on the periprosthetic bone mechanical environment under variable bone quality conditions.^12,13^ The presence of a femoral stem collar may also be biomechanically relevant in this context. In cementless THA, the collar is designed to rest on the calcar femorale and provide an additional proximal load-bearing interface. This may improve load transfer from the implant to the proximal femur, reduce excessive reliance on metaphyseal press-fit alone, and limit distal migration during early postoperative loading. Therefore, when stem–bone contact is reduced or primary fixation is potentially compromised, a collar may theoretically mitigate adverse changes in the periprosthetic mechanical environment.^14-16^

The primary aim of this study was therefore to investigate the influence of stem undersizing in THA performed via the DAA on periprosthetic bone biomechanics using FE modelling. By evaluating variations in implant sizing and design across a series of patient-specific computational models, we sought to elucidate how undersizing may alter load transfer patterns in the proximal femur and potentially contribute to mechanical pathways associated with implant failure. As a secondary objective, we investigated whether the presence of a femoral stem collar modifies load transfer patterns and mitigates the potential adverse mechanical effects associated with undersizing.

## Methods

To evaluate the biomechanical effects of stem undersizing in THA performed via the DAA, FE models of the full femur with implanted patient-specific femoral stems were developed. 3D femoral geometries were reconstructed from preoperative CT datasets (male 66 years, 80 kg; voxel size 0.68 mm × 0.68 mm × 0.6 mm) using semi-automatic segmentation techniques to obtain cancellous and cortical bone.

### Geometry model

The CT images were manually segmented using a custom application developed in the MATLAB 2012 environment (MathWorks, USA).^17^ Both the cortical and cancellous bone tissue were segmented separately, yielding 3D surface models of the femur exported in standard tessellation language (STL) format. STL files of two femoral stem designs used in THA (AMIStem-P and AMIStem-P Collared; Medacta International, Switzerland) were obtained by optical 3D scanning (Shining3D EinScan Pro HD; SHINING 3D Technology, Germany), providing accurate geometrical representations of the implant designs for subsequent virtual implantation and creation of computational models (Figure 1). All STL files were imported into SolidWorks (Dassault Systèmes, France) and converted into solid bodies. The patient-specific femoral model was selected because it provided high-quality CT data and representative proximal femoral anatomy suitable for controlled comparison of stem–bone fit conditions. The AMIStem-P stem was chosen because it is a cementless implant commonly used with the DAA and is available in both collarless and collared versions with comparable geometry, allowing assessment of collar presence while minimizing implant-design variability.

**Fig. 1:**
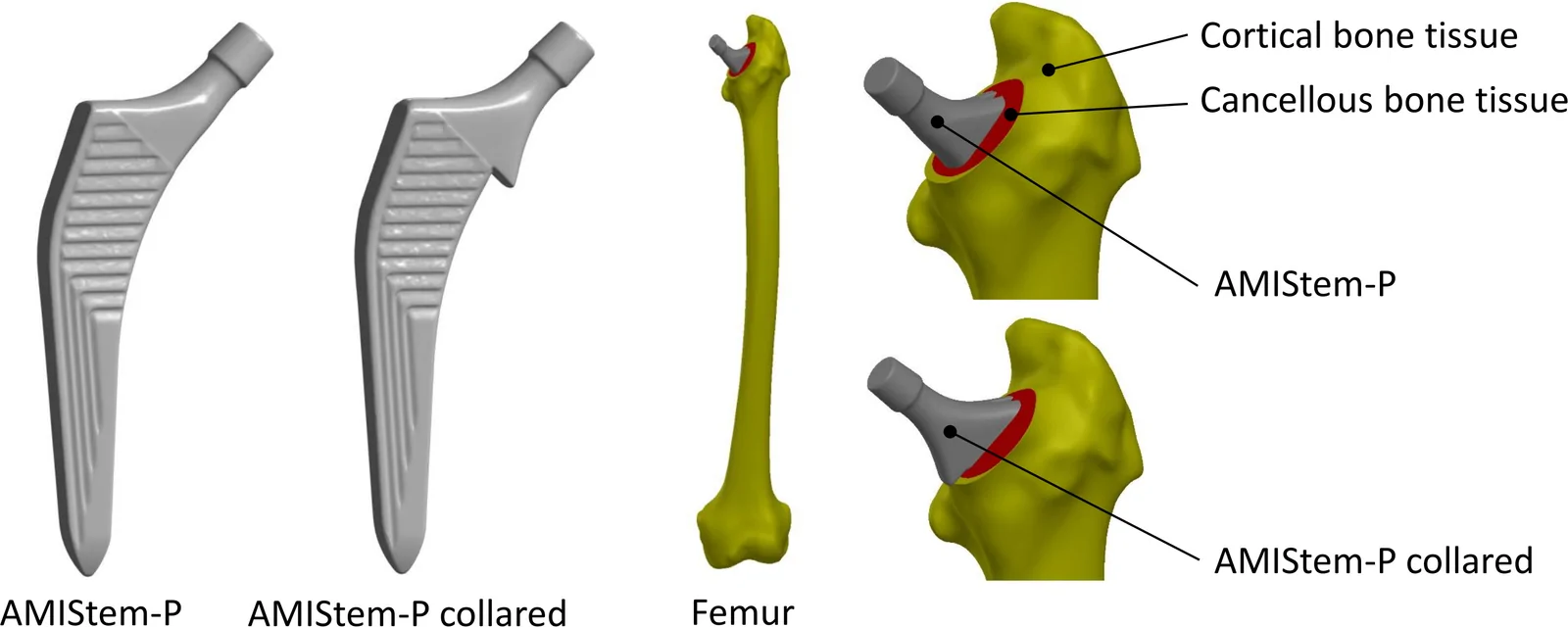
Patient-specific 3D geometrical model of the proximal femur with implanted femoral stems.

To investigate the effect of implant–bone fit, an undersized implantation computational model was first created in which the femoral stem exhibited reduced contact with the cortical bone. Subsequently, a modified femoral model was generated by isotropically scaling the bone geometry to 88% of its original size, while the stem geometry, position, and orientation were kept unchanged. This resulted in an adequate-fit configuration characterized by unchanged coronal alignment and insertion depth, increased metaphyseal engagement, greater proximal canal fill, and cortical support, particularly contact with the lateral femoral cortex. Consequently, all models remained directly comparable with respect to stem alignment and insertion depth, as illustrated in Figure 2.

**Fig. 2:**
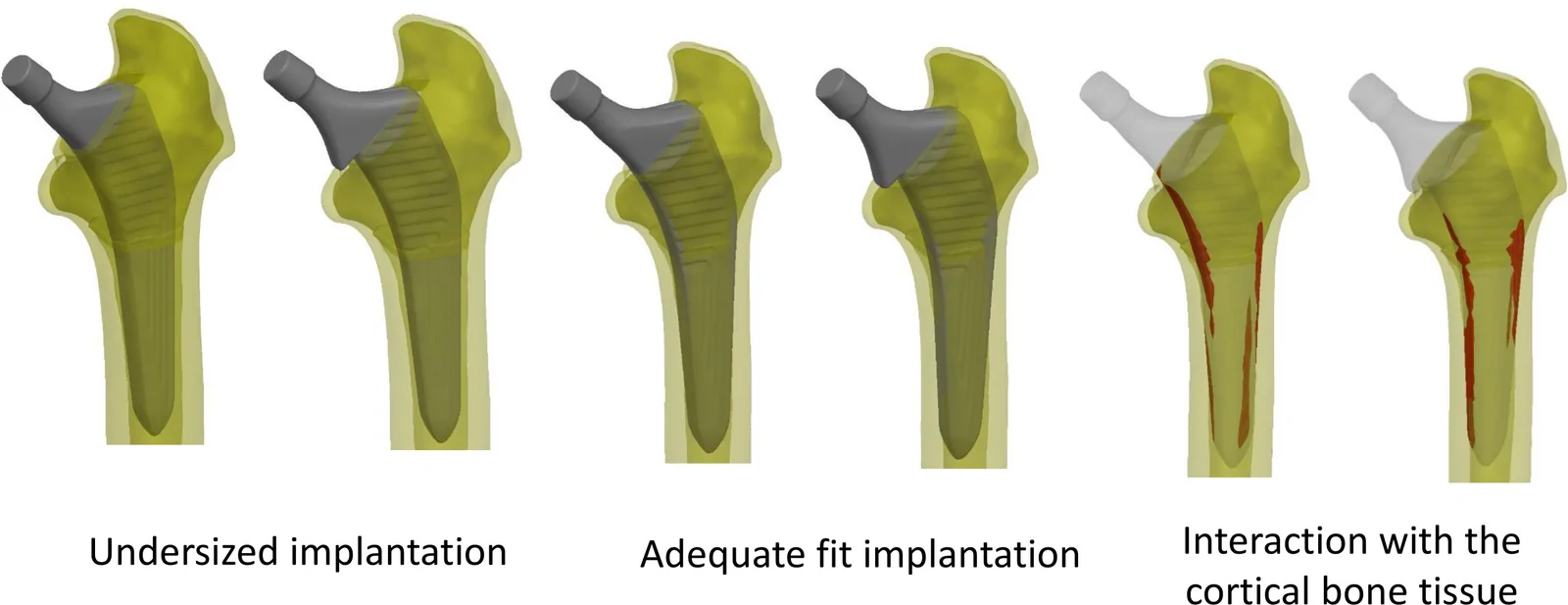
Variants of implantation representing the four geometrical configurations.

Final stem size and spatial positioning were established according to preoperative planning and intraoperative assessment of stability, in accordance with the orthopaedic surgeon’s experience and the manufacturer’s guidelines. As a result, four geometrical stem configurations were analyzed in this study, combining two sizing conditions and the presence or absence of a collar. The ‘Adequate Fit’ configuration corresponded to the standard, collarless AMIStem-P positioned within the modified (88%) femoral geometry, simulating an adequate stem–bone fit with cortical support. The ‘Adequate Fit–Collared’ configuration corresponded to the AMIStem-P Collared positioned within the same modified (88%) femoral geometry simulating an adequate stem–bone fit with cortical support. The ‘Undersized’ configuration corresponded to the collarless AMIStem-P positioned within the original femoral geometry simulating an undersized condition. The ‘Undersized–Collared’ configuration corresponded to the AMIStem-P Collared positioned within the original femoral geometry simulating an undersized condition. The undersized configuration represented reduced cortical engagement while maintaining the same stem position and alignment, allowing the isolated effect of reduced stem–bone contact to be evaluated.

### Computational mesh

The solid models of cortical and cancellous bone tissues with the implanted cementless femoral stem were assembled in SolidWorks and discretized in ANSYS Academic Research Mechanical, Release 25 R2 (Swanson Analysis, Inc., USA) using quadratic hexahedral and quadratic tetrahedral elements (SOLID186 and SOLID187, respectively). The contact interface between the femoral stem and the periprosthetic bone was modelled using contact elements CONTA174 and TARGE170, with a friction coefficient of 0.63.^18^ The mesh consisted of approximately three million elements. The global element size for cancellous bone tissue was 0.6 mm, and for the femoral stem and cortical bone tissue it was 2 mm, with local refinement to 0.8 mm on the defined contact surfaces (Figure 3).

**Fig. 3:**
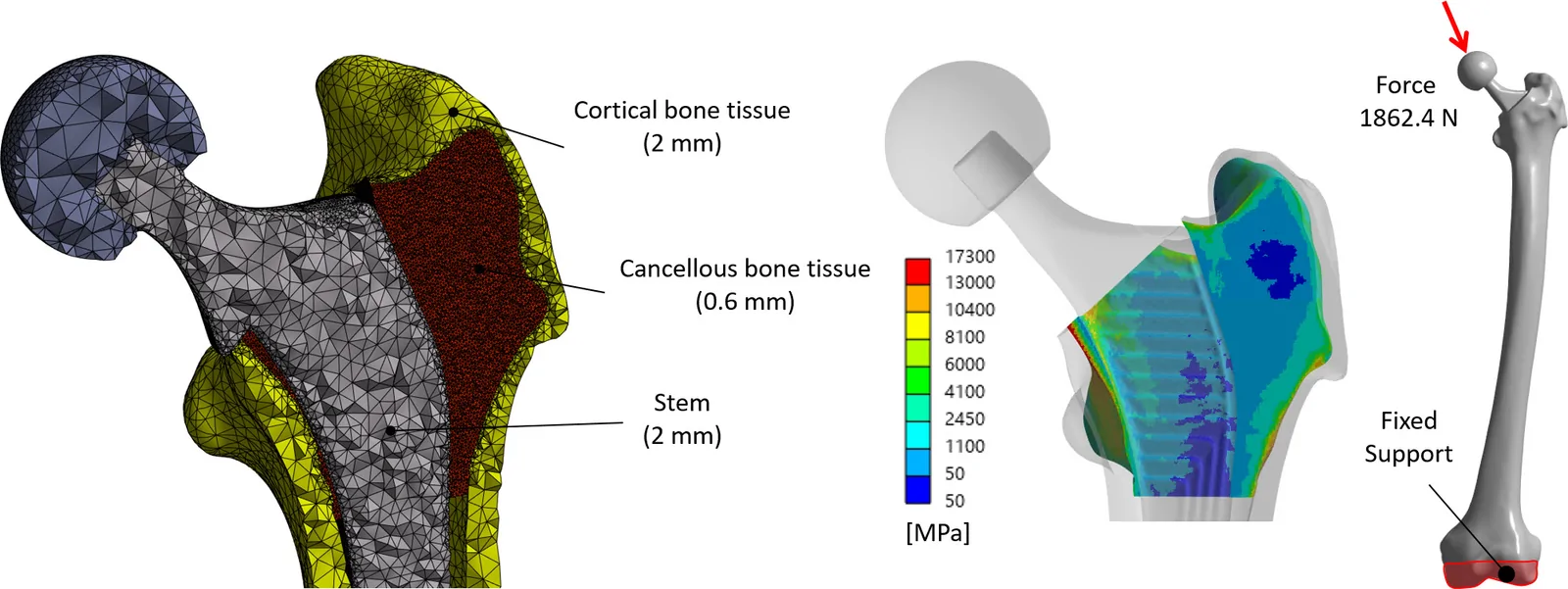
Finite element mesh of the proximal femur with implanted femoral stem, illustrating the discretization of cortical bone, cancellous bone, and implant components; spatial distribution of Young’s modulus within cancellous bone tissue; and applied boundary conditions and loading.

### Material model

All materials were assumed to be linearly elastic and isotropic. The cortical bone and femoral stem were modelled as homogeneous materials (E_cor_ = 17,300 MPa, μ_cor_ = 0.3; E_stem_ = 110,000 MPa; μ_stem_ = 0.3), whereas the cancellous bone tissue was assigned two density-dependent material variants (Table I).^18,19^ A reference material model representing 100% cancellous bone density was defined based on CT-derived relationships between Hounsfield units and Young’s modulus, reported previously (Figure 3).^20^ To simulate reduced bone quality, a second material variant with globally reduced stiffness corresponding to 90% of the reference cancellous bone density was created by proportionally decreasing the Young’s modulus values of the cancellous bone tissue.^21,22^

**Table I.** Material properties used in the finite element models.

Material	Young’s modulus, E (MPa)	Poisson’s ratio, μ (-)*	Reference
Cortical bone tissue	17,300	0.30	^19^
Femoral stem	110,000	0.30	^23^

* Unitless.

Thus, two bone density conditions were analyzed in the cementless femoral stem model: physiological cancellous bone density (100%) and reduced cancellous bone density (90%), while all other material properties remained unchanged.

### Constraints and loads

The distal femur was fully constrained in all degrees of freedom. Loading was applied as a resultant hip joint force acting on the prosthetic head and oriented to represent the peak loading phase during normal walking/single-leg support. The applied force of 1,862.4 N was oriented at angles of 106.9°, 79.3°, and 20.1° with respect to the positive mediolateral, anteroposterior, and proximodistal axes, respectively.^24,25^

### Biomechanical evaluation

In this study, four geometrical configurations were analyzed, combining two stem–bone fit conditions and the presence or absence of a collar. Each configuration was evaluated under two cancellous bone density conditions, resulting in eight FE models. The resulting computational models were used to evaluate the maximum shear strain in the periprosthetic bone, defined as the difference between the maximum and minimum principal strains and referred to throughout the paper as “strain intensity”.^17,26^ This evaluation approach is based on the Mechanostat hypothesis, which builds on Wolff’s law stating that strain stimuli in bone tissue influence its internal architecture.^27^ According to the Mechanostat concept, specific strain ranges may promote or inhibit bone modelling and remodelling. Equivalent (von Mises) stress was evaluated in the femoral stems.

## Results

### Periprosthetic strain intensity

Figure 4 illustrates regions with strain intensity exceeding 3,000 µε which, according to the mechanostat concept, corresponds to an overload zone of bone tissue. In the Undersized variant, a larger volume of bone tissue within the affected region was subjected to elevated strain levels corresponding to the overload zone. In contrast, the Adequate Fit configuration demonstrates a substantial reduction in both the extent and magnitude of overloaded regions. Compared with their collarless counterparts, the Undersized–Collared and Adequate Fit–Collared variants showed a reduced volume of periprosthetic bone exposed to overload-level strains. Overall, these findings highlight the significant influence of stem sizing and collar presence on the mechanical loading of the periprosthetic bone.

**Fig. 4:**
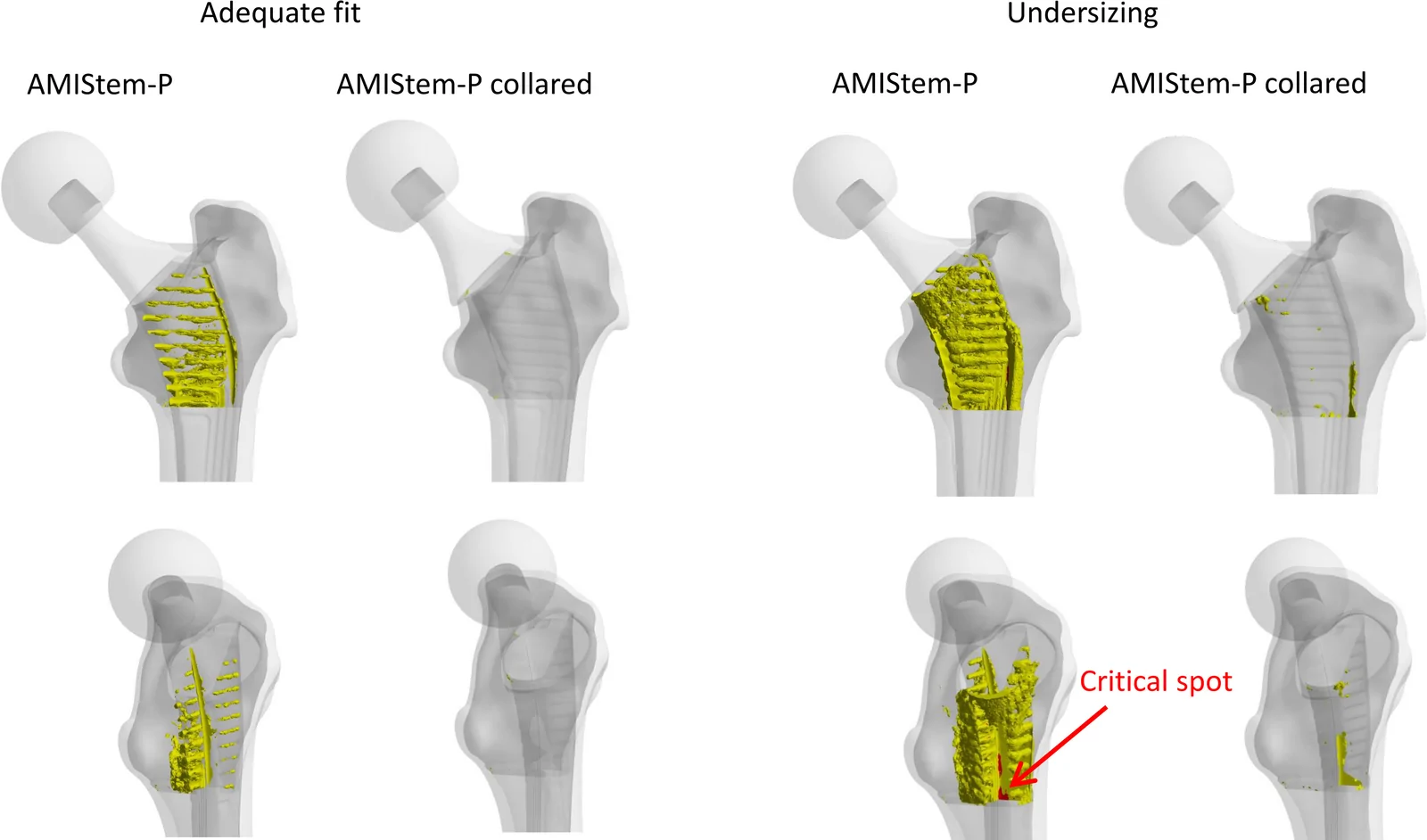
Distribution of periprosthetic cancellous bone strain intensity at reference bone density (100%) across the four configurations.

Figure 5 illustrates regions with strain intensity exceeding 3,000 µε. In this configuration, bone density was reduced by 10%. In the Undersized stem variant, cancellous bone demonstrated a pronounced increase in the volume of bone tissue exposed to overload-level strain.

**Fig. 5:**
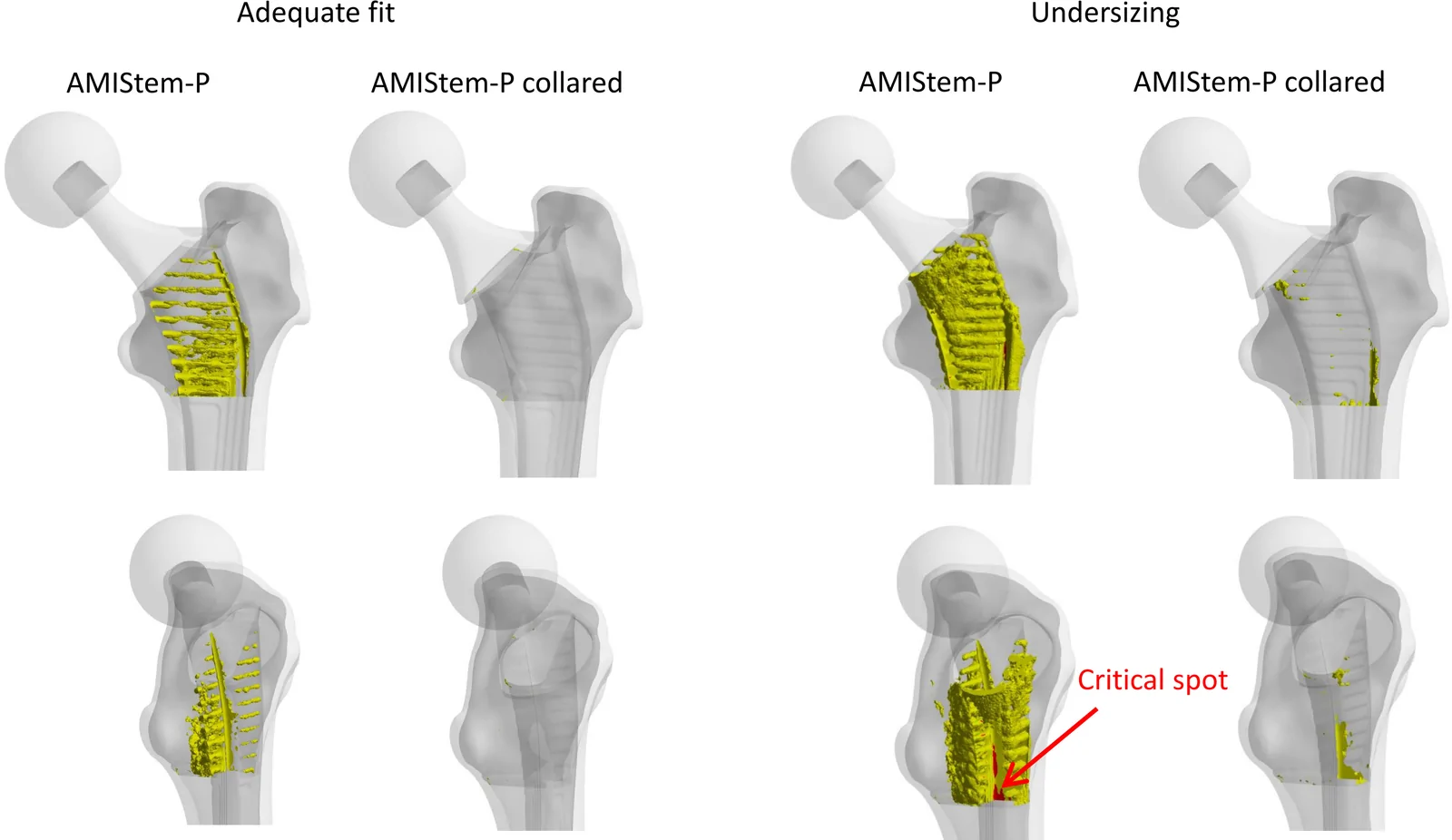
Distribution of periprosthetic cancellous bone strain intensity at reduced bone density (90%) across the four configurations.

Figure 6 summarizes the distribution of strain intensity within the cancellous bone for all evaluated configurations. Particular attention is directed to the yellow zone (> 3,000 µε), representing the overload region according to Frost’s mechanostat theory, and to the red zones ( > 25,000 µε), corresponding to potentially pathological strain levels.

**Fig. 6:**
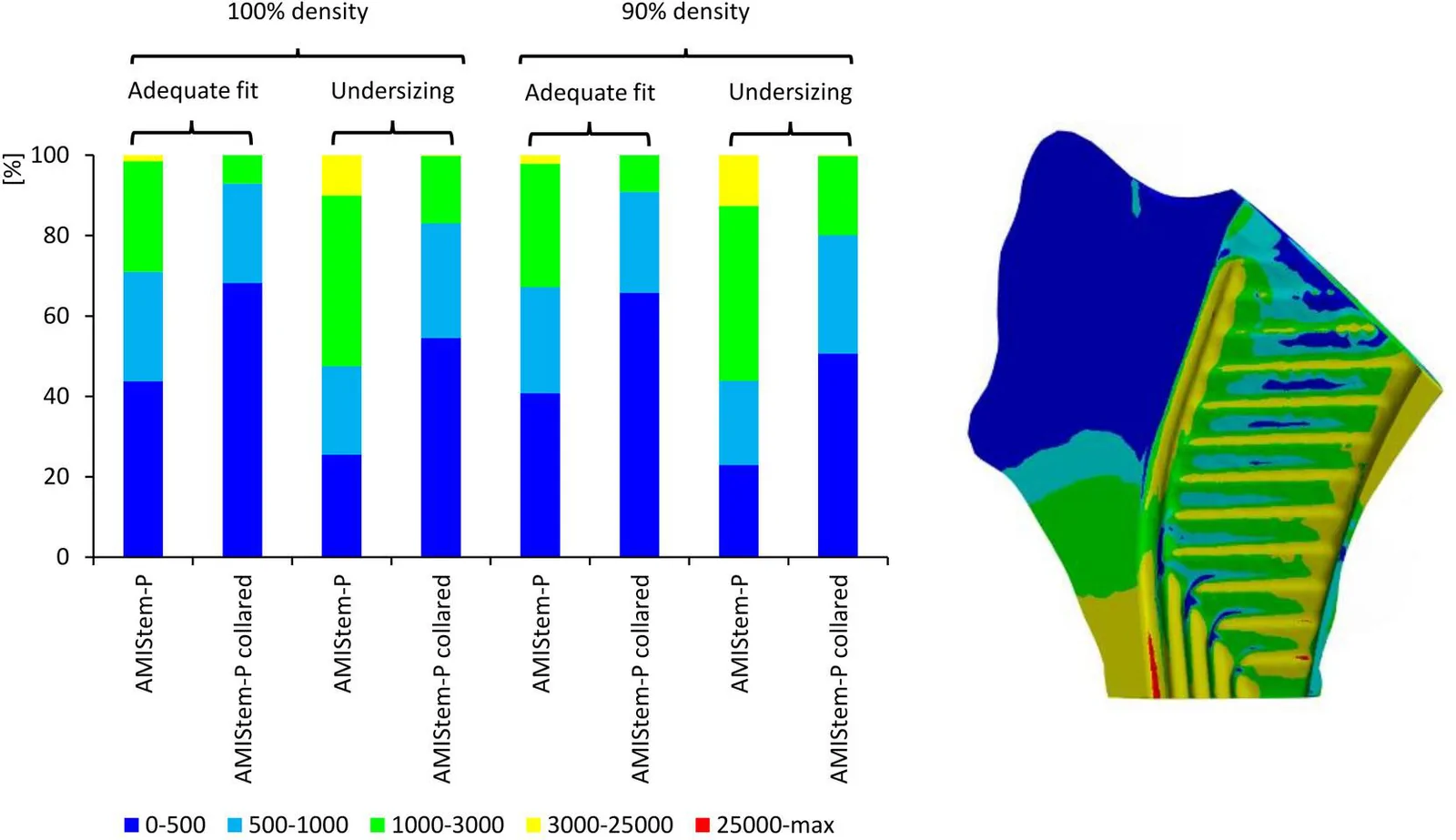
Percentage fraction distributions among mechanostat intervals, cancellous bone volume within predefined strain intervals for all model configurations, accompanied by an illustrative depiction of strain intensity distribution in the metaphyseal region.

In the Undersized configuration, the proportion of bone exposed to strains exceeding 3,000 µε is markedly increased compared with the Adequate Fit stem. Specifically, undersizing results in a substantial shift of bone volume from the physiological strain range (≤ 3,000 µε) toward the overload zone, indicating a deterioration of load distribution.

For comparative purposes, the worst-case scenario was selected, namely the Undersized configuration, and loading conditions were subsequently reduced to further evaluate the mechanical response of the periprosthetic bone (Figure 7).

**Fig. 7:**
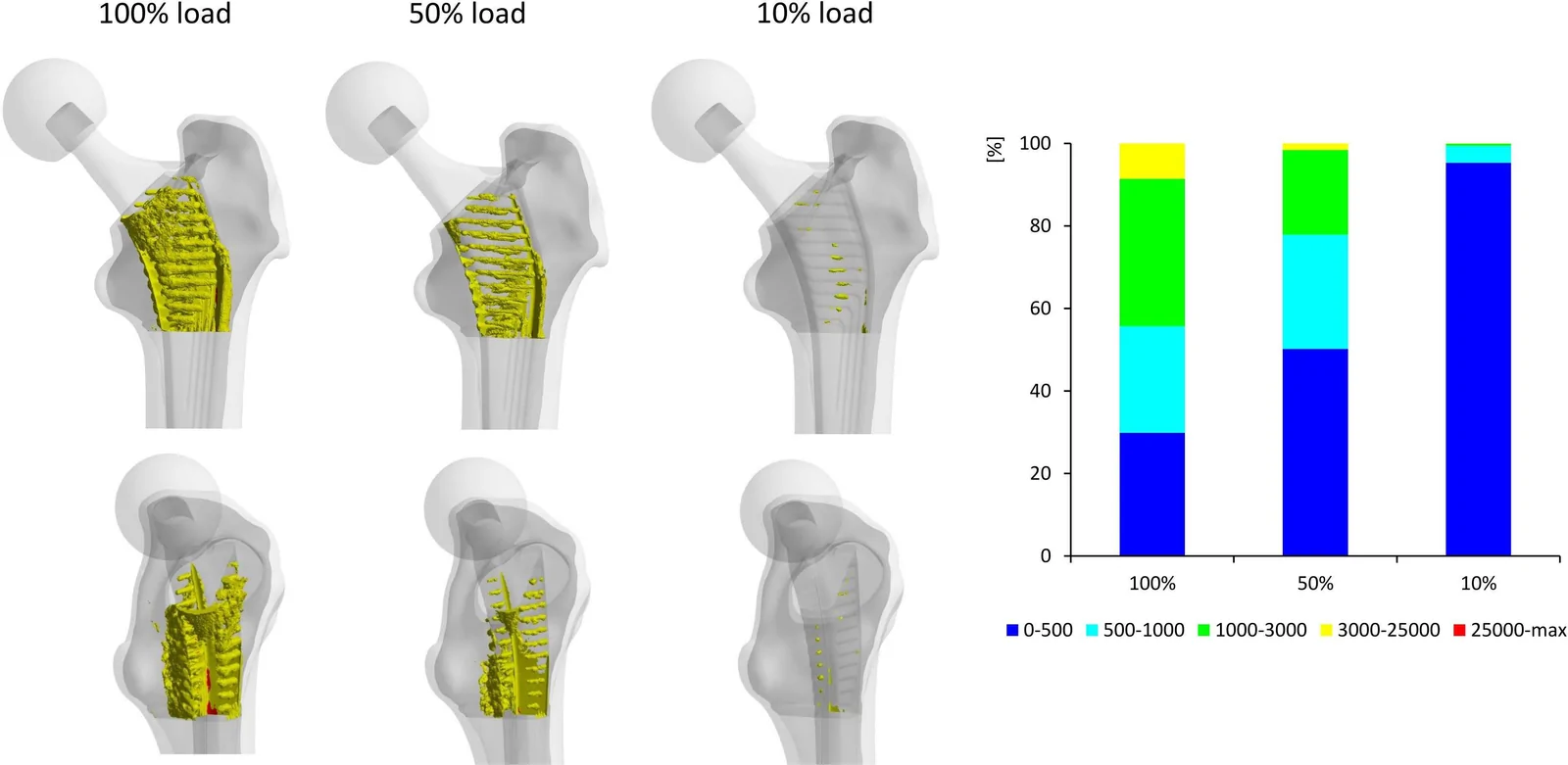
Percentage fraction distributions among mechanostat intervals. Periprosthetic cancellous bone strain intensity in the Undersized configuration with AMIStem-P under different loading conditions and the relative percentage of bone volume within predefined strain intervals.

### von Mises stress distribution in stem

The von Mises stress distribution within the stem illustrates changes in the loading pattern between configurations (Figure 8 and Figure 9). In the Undersized variant, stress is more concentrated in the neck region of the stem compared with the Adequate Fit configuration. Bone density had a negligible influence on the magnitude and distribution of stress within the implant.

**Fig. 8:**
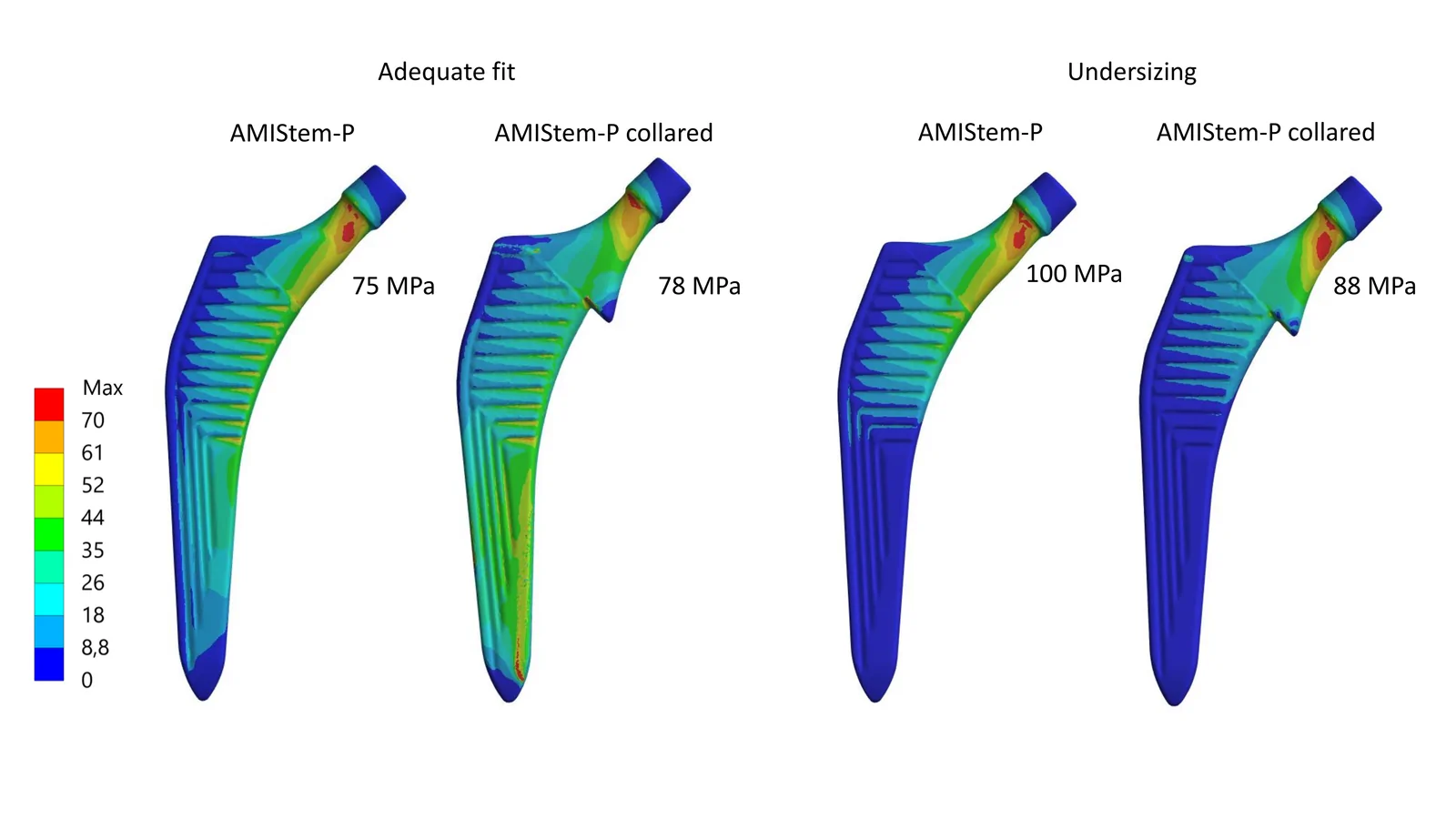
Distribution of von Mises stress within the femoral stem at reference bone density (100%) across the four geometrical configurations.

**Fig. 9:**
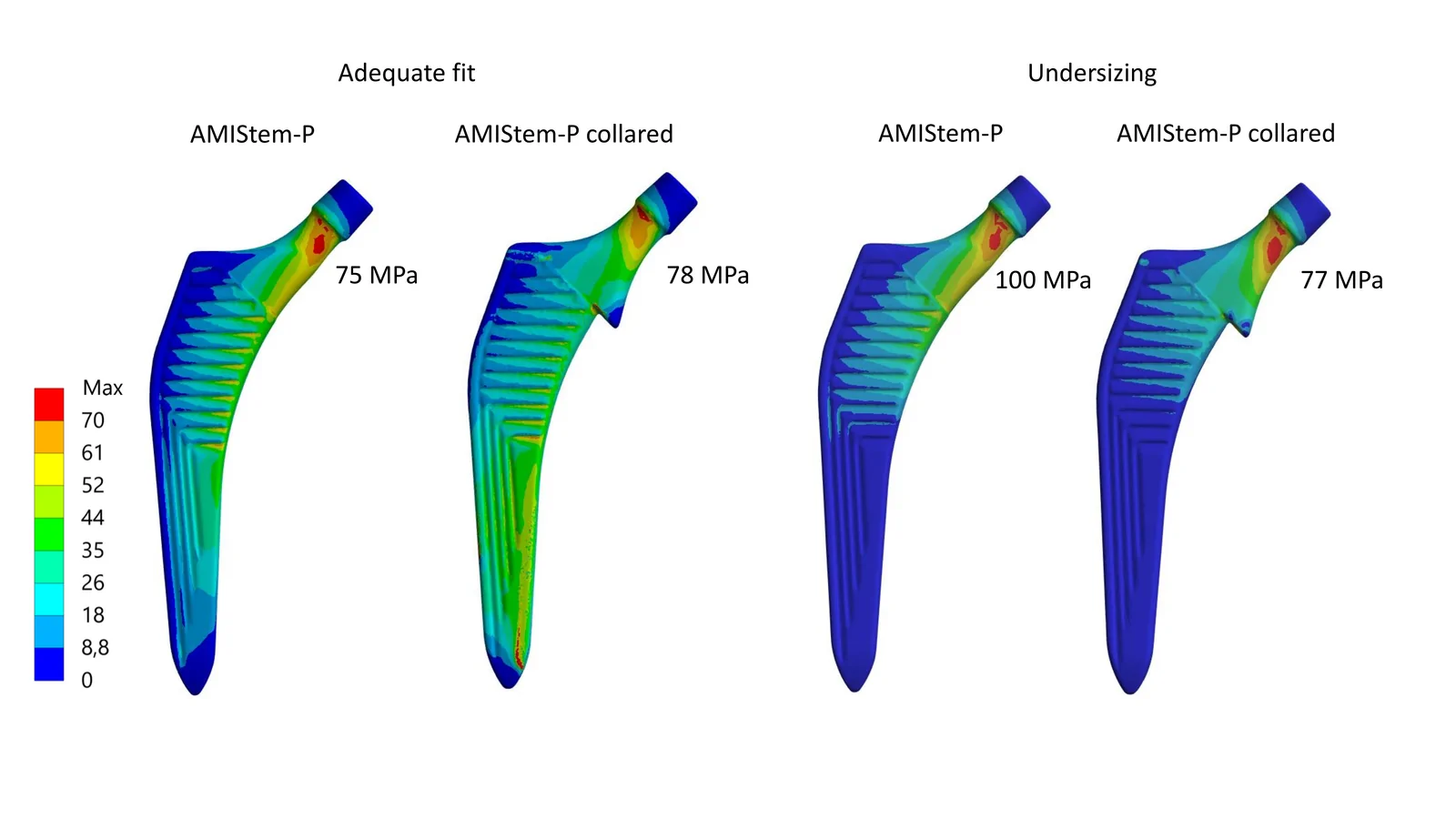
Distribution of von Mises stress within the femoral stem at reduced bone density (90%) across the four geometrical configurations.

The equivalent stress values were 80 to 100 MPa, which for titanium alloy are well below both the yield strength and the fatigue limit.^23^ Therefore, implant material failure is not expected to represent a mechanical concern under the evaluated loading conditions.

## Discussion

This study investigated the biomechanical consequences of femoral stem undersizing in THA performed through the DAA using patient-specific FE models of the femur. Eight FE models were analyzed, comprising four geometrical implantation configurations evaluated under two cancellous bone density conditions. The results demonstrated that the use of an undersized stem markedly increased the volume of bone subjected to elevated strain in the periprosthetic region, substantially enlarging the regions exceeding the mechanostat overload threshold (> 3,000 µε). In contrast, the Adequate Fit configuration substantially reduced the extent and magnitude of overloaded bone. The presence of a collar further improved load transfer patterns, reducing the proportion of cancellous bone exposed to elevated strain levels, particularly in the undersized configurations.

In the Undersized configuration, a markedly larger volume of bone was exposed to strain levels exceeding the mechanostat overload threshold, indicating deterioration of proximal load transfer. In contrast, when adequate stem–bone fit was achieved and the stem engaged the cortical bone, the extent of overloaded trabecular tissue decreased markedly. This finding highlights the importance of cortical support in redistributing load and limiting excessive strain within cancellous bone. These results are consistent with previous clinical and experimental studies emphasizing the importance of sufficient metaphyseal and cortical contact for primary stability in cementless THA. Clinical investigations have demonstrated that undersized stems show increased axial migration and reduced primary stability compared with adequately sized implants, most likely due to insufficient engagement with the lateral femoral cortex.^10,11,28,29^ Similarly, experimental and computational studies have reported greater micromotion and reduced resistance to subsidence when press-fit is inadequate.^13,30^ Together, these findings support the concept that insufficient stem–bone fit may compromise proximal load transfer and increase the mechanical demand on the periprosthetic trabecular bone, potentially contributing to early implant migration or suboptimal fixation. Although the undersized configuration may appear clinically acceptable visually, it represents a controlled reduction in cortical engagement. More severe clinical scenarios, such as varus insertion or markedly reduced canal fill, were not modelled and may produce greater biomechanical changes.

A potential strategy to mitigate the adverse mechanical consequences of stem undersizing is the use of a collared femoral stem. In the present study, the presence of a collar substantially reduced the volume of trabecular bone exposed to elevated strain levels, particularly in the undersized configurations. This effect can be explained by the mechanical support provided by the collar at the calcar femorale, where the implant rests against the cortical bone and thereby limits further distal migration of the stem. By providing an additional load-bearing interface in the proximal femur, the collar helps redistribute forces and reduces excessive strain within the periprosthetic cancellous bone. Previous clinical and experimental studies have also demonstrated the stabilizing effect of collared femoral stems. Biomechanical experiments have shown that collared stems provide greater immediate axial and rotational stability compared with collarless designs.^31^ Furthermore, recent systematic reviews have reported reduced early subsidence and lower revision rates for collared stems in total hip arthroplasty, suggesting that the collar may provide a protective effect against stem migration, particularly in technically challenging implantations.^14,15,32-34^ This mechanism may improve primary implant stability during early postoperative loading and reduce the risk of stem subsidence during weightbearing activities such as walking.

Although the simulations demonstrated increased strain levels in the periprosthetic trabecular bone in the Undersized configuration, critical strain levels associated with direct structural failure of bone (> 25,000 µε) occurred only locally and in limited regions. This suggests that, despite the increased mechanical demand on the periprosthetic bone, mechanical failure of the periprosthetic bone is unlikely under the evaluated loading conditions. Nevertheless, the presence of localized overload indicates that the mechanical environment may be suboptimal and therefore requires careful clinical consideration. One potential strategy to mitigate excessive strain is temporary reduction of postoperative loading. In the present study, simulated load reduction (assisted ambulation with crutches) resulted in a substantial decrease in periprosthetic bone strain, and pathological overload was no longer observed. These findings suggest that controlled or protected weightbearing during the early postoperative period may reduce mechanical loads around the implant and improve the mechanical environment for stable fixation, particularly in situations where primary fixation may be compromised, thereby potentially limiting implant migration or subsidence as reported in studies of cementless THA.^10,11,35^

This study has several limitations that should be considered when interpreting the results. First, the FE analysis was based on a single patient-specific femur and a single stem design. Therefore, the model does not fully capture the anatomical variability encountered in clinical practice, including differences in femoral morphology, canal geometry, cortical thickness, and bone quality. Accordingly, the findings should be interpreted as a comparative biomechanical assessment of the tested configurations rather than as directly generalizable to all patients or implant designs. Second, the loading conditions were simplified to a walking scenario and therefore do not reflect the full range of dynamic loads occurring during daily activities. Finally, the implant–bone interface was modelled to represent the early postoperative situation using a constant friction coefficient and did not account for biological processes such as osseointegration over time. Consequently, the results should not be interpreted as a direct prediction of clinical outcomes.

In summary, this FE analysis suggests that femoral stem undersizing may substantially increase the volume of periprosthetic trabecular bone exposed to strain levels associated with overload conditions. Achieving adequate stem–bone fit with cortical support was associated with a marked reduction in periprosthetic bone strain. The presence of a collar appeared to partially mitigate the adverse mechanical effects of undersizing by improving proximal load transfer. Reduced cancellous bone density resulted in a moderate additional increase in strain, although this effect was less pronounced than that of stem–bone fit.

## Data Availability

The data supporting the findings of this study are publicly available in the Zenodo repository at .

## References

[1] Galakatos GR Direct anterior total hip arthroplasty Mo Med 2018 115 6 537 541 30643349 PMC6312152

[2] Panichkul P Bavonratanavech S Arirachakaran A Kongtharvonskul J Comparative outcomes between collared versus collarless and short versus long stem of direct anterior approach total hip arthroplasty: a systematic review and indirect meta-analysis Eur J Orthop Surg Traumatol 2019 29 8 1693 1704 10.1007/s00590-019-02516-1 31363848

[3] Rivera F Leonardi F Evangelista A Pierannunzii L Risk of stem undersizing with direct anterior approach for total HIP arthroplasty Hip Int 2016 26 3 249 253 10.5301/hipint.5000337 27013489

[4] Australian Orthopaedic Association National Joint Replacement Registry (AOANJRR) Hip, Knee & Shoulder Arthroplasty: 2023 Annual Report Adelaide (SA) Australian Orthopaedic Association 2023 10.25310/YWQZ9375 https://aoanjrr.sahmri.com/documents/10180/1579982/AOA_NJRR_AR23.pdf/c3bcc83b-5590-e034-4ad8-802e4ad8bf5b?t=1695887126627

[5] Rele S O’Bryan E Holder C Lewis PL Di Bella C Collared cementless femoral components reduce the revision rates in primary total hip arthroplasty using the direct anterior approach: an Australian Orthopaedic Association National Joint Replacement Registry Study J Arthroplasty 2024 39 9 Suppl 2 S340 S346 10.1016/j.arth.2024.05.009 38735543

[6] Peuchot H Jacquet C Fabre-Aubrespy M et al. No benefit of direct anterior over posterolateral approach in total hip arthroplasty using dual-mobility acetabular component for femoral neck fracture Bone Joint J 2024 106-B 5 Supple B 133 138 10.1302/0301-620X.106B5.BJJ-2023-0832.R1 38688506

[7] Karunaseelan KJ Nasser R Jeffers JRT Cobb JP Optimal hip capsular release for joint exposure in hip resurfacing via the direct anterior approach Bone Joint J 2024 106-B 3 Supple A 59 66 10.1302/0301-620X.106B3.BJJ-2023-0592.R1 38423117

[8] Nambiar M Cheng TE Onggo JR et al. No difference in functional, radiographic, and survivorship outcomes between direct anterior or posterior approach THA: 5-year results of a randomized trial Clin Orthop Relat Res 2021 479 12 2621 2629 10.1097/CORR.0000000000001855 34237041 PMC8726547

[9] Sidhu VS Plaskos C Smith GH Pierrepont JW Solomon MI Femoral components implanted through a direct anterior approach have higher postoperative anteversion compared to those implanted through a posterior approach in total hip arthroplasty Bone Joint J 2025 107-B 5 Supple A 55 61 10.1302/0301-620X.107B5.BJJ-2024-1101.R2 40306718

[10] Konow T Glismann K Lampe F Ondruschka B Morlock MM Huber G Stem size and stem alignment affects periprosthetic fracture risk and primary stability in cementless total hip arthroplasty J Orthop Res 2024 42 4 829 836 10.1002/jor.25729 37971200

[11] Kutzner KP Freitag T Bieger R Defining “undersizing” in short-stem total hip arthroplasty: the importance of sufficient contact with the lateral femoral cortex Hip Int 2022 32 2 160 165 10.1177/1120700020940276 32644828 PMC8978469

[12] Hennicke NS Kluess D Sander M Influence of stem design parameters on periprosthetic femoral fractures examined by subject specific finite element analyses Med Eng Phys 2023 119 104032 10.1016/j.medengphy.2023.104032 37634909

[13] Abdul-Kadir MR Hansen U Klabunde R Lucas D Amis A Finite element modelling of primary hip stem stability: the effect of interference fit J Biomech 2008 41 3 587 594 10.1016/j.jbiomech.2007.10.009 18036531

[14] Kayani B Staats K Haddad FS The impact of a femoral stem collar on primary hip arthroplasty Bone Joint J 2024 106-B 9 879 883 10.1302/0301-620X.106B9.BJJ-2024-0353 39216852

[15] Inabathula A Allan DG Ryan B Horberg J Thuppal S Delfino K Reduced fractures and stem subsidence at six months with collared stems in direct anterior cementless total hip arthroplasty Orthop Procs 2025 107-B SUPP_11 52 10.1302/1358-992X.2025.11.052

[16] Hirasawa R Oinuma K Hagiwara S et al. Collared fully hydroxyapatite-coated femoral components reduce early periprosthetic femoral fractures in total hip arthroplasty with the direct anterior approach: a matched cohort study Bone Joint J 2025 107-B 10 1011 1019 10.1302/0301-620X.107B10.BJJ-2024-1494.R1 41027597

[17] Marcián P Borák L Zikmund T et al. On the limits of finite element models created from (micro)CT datasets and used in studies of bone-implant-related biomechanical problems J Mech Behav Biomed Mater 2021 117 104393 10.1016/j.jmbbm.2021.104393 33647729

[18] Meisterhans M Dimitriou D Fasser M-R Hoch A Jud L Zingg PO Influence of offset on osseointegration in cementless total hip arthroplasty: a finite element study J Orthop Res 2024 42 7 1566 1576 10.1002/jor.25808 38376065

[19] Grimal Q Haupert S Mitton D Vastel L Laugier P Assessment of cortical bone elasticity and strength: mechanical testing and ultrasound provide complementary data Med Eng Phys 2009 31 9 1140 1147 10.1016/j.medengphy.2009.07.011 19683957

[20] Dastgerdi AK Bavil AY Berninger MT et al. Effects of K-wire diameter and insertion angle on femoral bone medial closing-wedge osteotomies: a finite element study Sci Rep 2025 15 1 20116 10.1038/s41598-025-04260-5 40541981 PMC12181366

[21] Apostolopoulos V Boháč P Marcián P et al. Biomechanical comparison of all-polyethylene total knee replacement and its metal-backed equivalent on periprosthetic tibia using the finite element method J Orthop Surg Res 2024 19 1 153 10.1186/s13018-024-04631-0 38396020 PMC10893603

[22] Rungruangbaiyok C Azari F van Lenthe GH Vander Sloten J Tangtrakulwanich B Chatpun S Finite element investigation of fracture risk under postero-anterior mobilization on a lumbar bone in elderly with and without osteoporosis J Med Biol Eng 2021 41 3 285 294 10.1007/s40846-021-00607-1

[23] Niinomi M Mechanical properties of biomedical titanium alloys Mater Sci Eng A 1998 243 1–2 231 236 10.1016/S0921-5093(97)00806-X

[24] Bergmann G Deuretzbacher G Heller M et al. Hip contact forces and gait patterns from routine activities J Biomech 2001 34 7 859 871 10.1016/s0021-9290(01)00040-9 11410170

[25] Soliman MM Mahmud S Chowdhury MEH et al. Stress transfer, stress shielding, and micro-movement analysis in the implanted femoral bone region with various hip implant stem materials: a finite element approach Materials Today Communications 2024 41 110932 10.1016/j.mtcomm.2024.110932

[26] Suzuki N Aoki K Marcián P Borák L Wakabayashi N A threshold of mechanical strain intensity for the direct activation of osteoblast function exists in a murine maxilla loading model Biomech Model Mechanobiol 2016 15 5 1091 1100 10.1007/s10237-015-0746-1 26578077

[27] Frost HM A 2003 update of bone physiology and Wolff’s Law for clinicians Angle Orthod 2004 74 1 3 15 10.1043/0003-3219(2004)0742.0.CO;2 15038485

[28] Suksathien Y Chuvanichanon P Tippimanchai T Sueajui J Insufficient lateral stem contact is an influencing factor for significant subsidence in cementless short stem total hip arthroplasty World J Orthop 2022 13 5 444 453 10.5312/wjo.v13.i5.444 35633743 PMC9124996

[29] Lacko M Schreierová D Čellár R Vaško G Bone remodelling in the proximal femur after uncemented total hip arthroplasty in patients with osteoporosis Acta Chir Orthop Traumatol Cech 2015 82 6 430 436 26787184

[30] Reiber M Bensel F Becker N Budde S Nackenhorst U Finite element analysis of stem migration after total hip replacement Biomech Model Mechanobiol 2025 24 5 1619 1631 10.1007/s10237-025-01985-0 40824611 PMC12454594

[31] Demey G Fary C Lustig S Neyret P si Selmi TA Does a collar improve the immediate stability of uncemented femoral hip stems in total hip arthroplasty? A bilateral comparative cadaver study J Arthroplasty 2011 26 8 1549 1555 10.1016/j.arth.2011.03.030 21570801

[32] Nerys-Figueroa J Parsa A Curley A Charif S Domb BG Schinsky MF Slightly reduced early subsidence with similar outcomes and complications rate in collared stems - a systematic review of randomized clinical trials J Orthop 2024 50 170 176 10.1016/j.jor.2024.01.013 38328796 PMC10845209

[33] Ait-Si-Selmi T Vidalain J-P Ramos-Pascual S et al. Outcomes of total hip arthroplasty using collared versus collarless uncemented HA-coated stems: a meta-analysis EFORT Open Rev 2024 9 4 264 275 10.1530/EOR-22-0091 38579748 PMC11044085

[34] Giovanoulis V Kenanidis E Aïm F et al. Collared versus collarless hydroxyapatite-coated stems for primary cementless total hip arthroplasty; a systematic review of comparative studies. Is there any difference in survival, functional, and radiographic outcomes? SICOT J 2024 10 8 10.1051/sicotj/2024003 38358293 PMC10868518

[35] Huang L Han W Qi W et al. Early unrestricted vs. partial weight bearing after uncemented total hip arthroplasty: a systematic review and meta-analysis Front Surg 2023 10 1225649 10.3389/fsurg.2023.1225649 38033533 PMC10684916

